# Association of Glyburide and Subcutaneous Insulin With Perinatal Complications Among Women With Gestational Diabetes

**DOI:** 10.1001/jamanetworkopen.2022.5026

**Published:** 2022-03-31

**Authors:** Monique M. Hedderson, Sylvia E. Badon, Noel Pimentel, Fei Xu, Anne Regenstein, Assiamira Ferrara, Romain Neugebauer

**Affiliations:** 1 Division of Research, Kaiser Permanente Northern California, Oakland; 2 Kaiser Permanente Bernard J. Tyson School of Medicine, Pasadena, California

## Abstract

**Importance:**

Nearly 30% of individuals with gestational diabetes (GDM) do not achieve glycemic control with lifestyle modification alone and require medication treatment. Oral agents, such as glyburide, have several advantages over insulin for the treatment of GDM, including greater patient acceptance; however, the effectiveness of glyburide for the treatment of GDM remains controversial.

**Objective:**

To compare the perinatal and neonatal outcomes associated with glyburide vs insulin using causal inference methods in a clinical setting with information on glycemic control.

**Design, Setting, and Participants:**

The population-based cohort study included patients with GDM who required medication treatment from 2007 to 2017 in Kaiser Permanente Northern California. Machine learning and rigorous casual inference methods with time-varying exposures were used to evaluate associations of exposure to glyburide vs insulin with perinatal outcomes. Data analysis was conducted from March 2018 to July 2017.

**Exposures:**

Time-varying exposure to glyburide vs insulin during pregnancy.

**Main Outcomes and Measures:**

Outcomes evaluated separately included neonatal hypoglycemia, jaundice, shoulder dystocia, respiratory distress syndrome (RDS), neonatal intensive care unit (NICU) admission, size-for–gestational age, and cesarean delivery. Inverse probability weighting (IPW) estimation was used to separately compare perinatal outcomes between those initiating glyburide and insulin. This approach was combined with Super Learning for propensity score estimation to account for both baseline and time-dependent confounding in both per-protocol (primary) and intention-to-treat (secondary) analyses to evaluate sustained exposure to the same therapy.

**Results:**

From 2007 to 2017, 11 321 patients with GDM (mean [SD] age, 32.9 [4.9] years) initiated glyburide or insulin during pregnancy. In multivariate models, the risk of neonatal respiratory distress was 2.03 (95% CI, 0.13-3.92) per 100 births lower and the risk of NICU admission was 3.32 (95% CI, 0.20-6.45) per 100 births lower after continuous exposure to glyburide compared with insulin. There were no statistically significant differences in glyburide vs insulin initiation in risk for neonatal hypoglycemia (0.85 [95% CI, −1.17 to 2.86] per 100 births), jaundice (0.02 [95% CI, −1.46 to 1.51] per 100 births), shoulder dystocia (−1.05 [95% CI, −2.71 to 0.62] per 100 births), or large-for–gestational age categories (−2.75 [95% CI, −6.31 to 0.80] per 100 births).

**Conclusions and Relevance:**

Using data from a clinical setting and contemporary causal inference methods, our findings do not provide evidence of a difference in the outcomes examined between patients with GDM initiating glyburide compared with those initiating insulin.

## Key Points

**Question:**

Are glyburide and subcutaneous insulin use associated with different rates of perinatal complications among individuals with gestational diabetes?

**Findings:**

In this cohort study of 11 321 patients with gestational diabetes, the use of glyburide vs insulin was associated with similar rates of infant hypoglycemia, hyperbilirubinemia, appropriate size-for–gestational age, and cesarean delivery as well as significantly lower rates of infant neonatal intensive care unit admission after adjusting for baseline and time-varying covariates.

**Meaning:**

These findings do not provide evidence of a difference in the outcomes examined among patients with gestational diabetes initiating glyburide compared with those initiating insulin.

## Teaser

This cohort study compares the perinatal outcomes associated with initiating glyburide vs insulin among individuals with gestational diabetes.

## Introduction

Gestational diabetes (GDM) is among the most common pregnancy complications, affecting as many as 10% of pregnancies in the United States.^1^ GDM is associated with maternal morbidity, such as cesarean delivery, and with neonatal hypoglycemia, large-for–gestational age infants, and neonatal intensive care unit (NICU) admission.^2^ A key prevention strategy for reducing risk of perinatal complications associated with GDM is helping patients achieve optimal glycemic control.^3^ Medical Nutritional Therapy (MNT), including diet, physical activity, and home glucose monitoring, is the recommended first-line treatment for GDM. However, 30% to 50% of individuals with GDM require the addition of pharmacological therapy to achieve optimal glycemic control.^4^

Historically, both insulin and the oral hypoglycemic agent glyburide were recommended for the treatment of GDM.^5^ Since oral agents such as glyburide have several advantages over insulin for the treatment of GDM, including greater ease of use, greater acceptance among patients, and lower cost,^6^ glyburide became widely used for treatment of GDM in the United States in the early 2000s.^7^ However, more recently controversy has arisen as to the safety and efficacy of glyburide for the treatment of GDM. A recent meta-analysis of 24 studies comparing perinatal outcomes between glyburide and insulin showed that while glyburide was associated with lower risk of cesarean delivery, it was also associated with greater risk of neonatal hypoglycemia and longer duration of NICU admission.^8^ A limitation of previous randomized clinical trials comparing glyburide and insulin is that they have not accounted for changes in GDM medication after initial medication initiation and glyburide not being dosed according to its pharmacokinetic properties.^9^ In addition, previous observational studies have often not accounted for GDM severity or duration of use, which may explain differences in adverse perinatal outcomes between glyburide and insulin treatment.

The objective of this study was to emulate per-protocol (PP) and intention-to-treat (ITT) analyses of a conceptual randomized trial^10^ for comparing the association of sustained exposure to glyburide and insulin as first-line medication treatment for GDM with several adverse perinatal outcomes using observational data a clinical setting.

## Methods

### Study Setting

This study was conducted within Kaiser Permanente Northern California (KPNC), an integrated health care delivery system that provides medical care for about one-third of the Northern California population, including more than 40 000 deliveries per year. KPNC members are representative of the underlying population of this region.^11^

Within KPNC, 94% of pregnancies are screened for GDM,^12^ and 97% of patients with GDM are treated through a Regional Perinatal Service Center (RPSC), a telephone-based centralized nurse-based management program. Standard practice for GDM diagnosis is a 2-step approach (a 50-g, 1-hour glucose challenge test [GCT] followed by a diagnostic 100-g, 3-hour oral glucose tolerance test [OGTT]) and diagnosed according to the Carpenter and Coustan criteria.^13,14^ Some patients with a 50-g GCT of 180 mg/dL or greater and risk factors for GDM (ie, history of GDM or obesity) are also diagnosed with GDM. Patients with GDM are initially treated with MNT and home glucose monitoring. Typically, patients who have uncontrolled glucose levels for 2 weeks after starting on MNT are switched to the first-line medication therapy, which in KPNC during the study period was most often glyburide. If patients continue having uncontrolled blood glucose levels after initiating glyburide therapy, they are switched to insulin therapy. Some patients are initially started on insulin due to patient or clinician preference.

This study was approved by the Kaiser Permanente Northern California institutional review board, and a waiver of informed consent was granted because the research involved minimal risk to the patients. We followed the Strengthening the Reporting of Observational Studies in Epidemiology (STROBE) guidelines.^15^

### Study Population

All data were extracted from the electronic health record using structured query language. We identified all singleton live births with initiation of medication therapy with either glyburide or insulin between 2007 and 2017. The date of first insulin or glyburide dispensation (index date) defined the start of follow-up.

### Exposures

Prescription fills for glucose-lowering medications including glyburide and insulin (rapid-, short-, intermediate-, and long-acting) during a GDM pregnancy were identified using the KPNC prescription medication database. Intervals of medication exposure were created based on the date the prescription was filled and the total number of days of supply. A categorical exposure variable was created at each 7-day interval with the following possible values: glyburide, insulin, not exposed to glucose-lowering medications, glyburide and other therapy (metformin or glipizide), insulin and other therapy (metformin or glipizide), and other therapy (metformin or glipizide).

### Outcomes

Each outcome was assessed separately. Perinatal and neonatal outcomes included neonatal hypoglycemia (plasma glucose <40 mg/dL [to convert to millimoles per liter, multiply by 0.0555]), jaundice (bilirubin levels ≥20 mg/dL [to convert to micromoles per liter, multiply by 17.104]), shoulder dystocia (*International Classification of Diseases *[*ICD*] codes), respiratory distress (*ICD* codes), NICU admission from our Neonatal Minimum Data Set,^16^ and birthweight-for–gestational age category (defined using KPNC-specific race and ethnicity and gestational age–specific birth weight curves).^17^ Small-for–gestational age birth weight was defined as birth weight–for–gestational age below the 10th percentile; large-for–gestational age birth weight was defined as birth weight–for–gestational age above the 90th percentile of these birth weight curves. Cesarean delivery was based on *ICD* and procedure codes.

### Covariates

#### Glycemic Control Data

Data on capillary glucose values were obtained from self-monitoring data on fasting and 1-hour postprandial measurements. Patients with GDM are provided a standard glucometer and are recommended to self-monitor their glucose in the morning, while fasting, and 1 hour after breakfast, lunch, and dinner. Glucose measurements are verbally reported to the RPSC during weekly telephone calls, and data are manually recorded in a clinical database. Optimal glycemic control is defined as 80% or more of all capillary glucose measurements meeting the targets recommended in this clinical setting: less than 95 mg/dL for fasting and less than 140 mg/dL for 1 hour after meals. We created a time-varying optimal glycemic control variable based on all glucose measurements (fasting, 1 hour after meals) in a 7-day interval; if 80% of these measurements met their corresponding targets, individuals were considered to have achieved optimal glycemic control within that specific 7-day interval.

#### Other Covariates

We identified a comprehensive list of covariates (eTable 1 in the Supplement) potentially affecting medication therapy used for GDM treatment and outcomes. These included maternal age, medical history, smoking and substance use during pregnancy, GDM screening glucose values as a marker of severity, and glycemic control. Self-reported race (American Indian or Alaska Native, Asian or Pacific Islander, Black, White, and multiracial) and ethnicity (Hispanic and non-Hispanic) was captured in the outpatient setting, hospital admission, and/or at the time of enrollment in the health plan and all patients self-identified as Hispanic ethnicity, regardless of race, were categorized as Hispanic. We included race and ethnicity as covariates given that both are associated with medication use and with perinatal outcomes of interest.

### Statistical Analysis

We aimed to emulate ITT^18^ and PP^19^ analyses of a conceptual trial in which eligible pregnant individuals who consented to initiate insulin or glyburide for GDM during their pregnancy occurring between 2007 and 2017 would be randomized at the time of drug dispensing to either sustained therapy with insulin or glyburide through the end of their pregnancy (when all outcomes would be collected). In the PP analysis (primary), we aim to estimate the effect of sustained exposure to each glucose-lowering therapy, while in the ITT analysis, we only aim to estimate the effect of initiating each of these therapies at the start of follow-up (participants are free to change therapies or stop glucose-lowering therapy entirely after initiation of each therapy). To be eligible, individuals need a diagnosis of GDM and no prior diagnosis of type 2 diabetes. Additional inclusion and exclusion criteria are described in the Figure. Mean pregnancy outcomes between treatment groups and their differences are what we aimed to estimate using observational data. For each outcome considered, a separate analytic data set was constructed.^20^ Measurements on exposure, outcome, and covariates were updated every 7 days between the index date and end of pregnancy.

**Figure.**
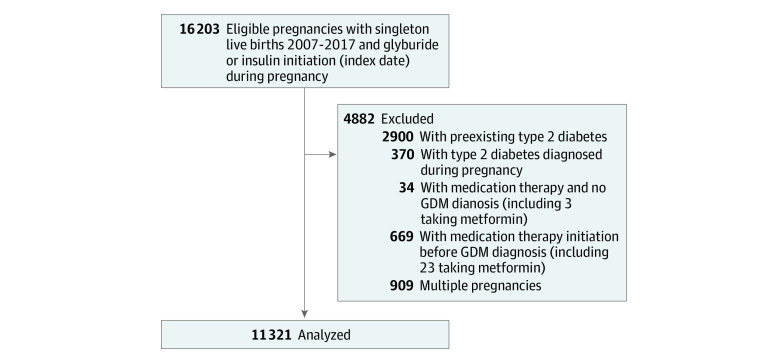
Analytic Cohort Creation for Comparison of Glyburide vs Insulin Treatment on Subsequent Perinatal Outcomes GDM indicates gestational diabetes.

We fitted a linear marginal structural model with inverse probability weighting^21,22^ to estimate the counterfactual risks and risk differences defined by the conceptual trial above. Propensity scores, which define the inverse probability weights, were used to adjust for selected potential time-independent confounders in ITT analyses (eTable 7 in the Supplement) and for both time-independent and time-dependent potential confounders in PP analyses (eTables 3-6 in the Supplement). In ITT analyses, the point-treatment binary exposure is defined by the medication dispensed on index date. In PP analyses, the exposure at each 7-day of follow-up is categorical with 6 levels. Table 1 and our prior work^23^ provide more details on our approach to propensity score estimation with categorical exposure with more than 2 levels. The propensity scores associated with these 2 distinct exposure definitions were estimated using a data-adaptive estimation known as Super Learning.^24,25,26^ Super Learning is an ensemble learning method that was used for both adapting the covariate adjustment set that best estimates the propensity scores (covariate selection) and allowing for complex, nonlinear associations between covariates and exposure. In short, Super Learning consists of combining estimated values from various candidate learners through a weighted average. The selection of the optimal combination of learners is based on cross-validation to protect against overfitting such that the resulting learner (called super learner) performs asymptotically as well or better than any of the candidate learners considered. As described in eTables 3 to 7 in the Supplement, each propensity score was estimated using the following 9 learner types, each fitted 5 times using a distinct subset of covariates defined by 5 screeners: a main-term-only logistic model (glm), linear splines with tensor products^27^ (polyclass), random forests^13^ (rngr), extreme gradient boosting regression^28,29^ (xgb), generalized additive model (gam), lasso regression (glmnet), bayesian additive regression tree (bart),^30^ a logistic model with all 2-way interaction terms (glm.int), and a neural network regression with a single hidden layer (nnet). The 4 screeners automatically select the first 5, 10, 20, and 30 covariates most associated with the exposure. The fifth screener selects all covariates in eTable 1 in the Supplement. Continuous covariates (eTable 1 in the Supplement) were considered “as is” by each learner, and screener combination was also discretized using the cutoffs provided in eTable 2 in the Supplement. The dummy variables resulting from discretization were also considered as separate factors by each learner and screener combination. All inverse probability weights were stabilized. No weight truncation^31,32^ was implemented owing to the very compact distribution of the weights with no inverse property weights larger than 3.82 and 5.75 in the ITT and PP analyses, respectively.

**Table 1.** Characteristics of 11 321 Pregnant Individuals With GDM Requiring Medication Therapy by Initial Medication Type^a^

Characteristic	Participants by drug initiated, No. (%)	
	Glyburide (n = 10 249)	Insulin (n = 1072)
Maternal age, mean (SD), y	32.9 (4.9)	32.4 (5.2)
Race and ethnicity		
American Indian, Alaska Native, multiracial, unknown	615 (6.0)	70 (6.5)
Asian or Pacific Islander	3664 (35.8)	277 (25.8)
Hispanic	2891 (28.2)	324 (30.2)
Non-Hispanic Black	525 (5.1)	58 (5.4)
Non-Hispanic White	2535 (24.7)	342 (31.9)
Missing	19 (0.2)	1 (0.1)
Median household income in census tract of residence, mean (SD), $	74 163 (29 978)	69 859 (28 857)
Medical history at index date		
Nulliparous	4029 (39.3)	413 (38.5)
Prepregnancy BMI		
Mean (SD)	30.8 (7.2)	32.8 (8.0)
Underweight, <18.5	65 (0.6)	4 (0.4)
Normal weight, 18.5-24.9	2190 (21.4)	156 (14.6)
Overweight, 25.0-29.9	2708 (26.4)	235 (21.9)
Obese, ≥30.0	4741 (46.3)	568 (53.0)
Missing	545 (5.3)	109 (10.2)
Depression	660 (6.4)	73 (6.8)
Hypertension^b^		
Prepregnancy	594 (5.8)	72 (6.7)
Gestational	92 (0.9)	13 (1.2)
Preeclampsia^b^	52 (0.5)	4 (0.4)
Antihypertensive medication use	433 (4.2)	55 (5.1)
Gestational weight gain to index date, mean (SD), kg/wk	0.23 (0.21)	0.23 (0.26)
Blood pressure at index, mean (SD), mm Hg		
Systolic	114.6 (12.5)	117.3 (13.0)
Diastolic	67.6 (9.3)	69.5 (9.5)
Behaviors during pregnancy at index date		
Smoked	320 (3.1)	46 (4.3)
Used alcohol	747 (7.3)	93 (8.7)
Used illicit drugs	145 (1.4)	21 (2.0)
GDM diagnosis^c^		
Gestational age at GDM diagnosis, mean (SD), wk	23.4 (7.1)	23.0 (8.1)
50-g GCT screening value, mean (SD), mg/dL	173.4 (26.4)	196.3 (57.2)
Glycemic control		
Any hypoglycemic episodes	3 (<0.1)	0
GDM treatment course, mean (SD)		
Gestational age at index	28.3 (6.4)	26.6 (7.9)
Time since GDM diagnosis at index, wk	5.0 (4.1)	3.6 (3.9)
Medication course		
Continuously on initial medication	8845 (86.3)	707 (66.0)
Switched to		
No pharmacotherapy	747 (7.3)	331 (30.9)
Combination therapy including glyburide^d^	646 (6.3)	9 (0.8)
Insulin	10 (0.1)	NA
Glyburide	NA	0
Other therapy (metformin or glipizide)	1 (<0.1)	1 (<0.1)
Combination therapy including insulin^e^	0	24 (2.2)
Time from index to any switch in medication therapy, median (IQR), wk	5.0 (5.0-13.0)	5.0 (5.0-5.0)
Duration of follow-up, mean (SD), wk	9.1 (6.4)	10.5 (7.8)
Gestational age at delivery	38.3 (1.6)	38.0 (1.9)
Macrosomia	1325 (12.9)	168 (15.7)

Abbreviations: BMI, body mass index (calculated as weight in kilograms divided by height in meters squared); GCT, glucose challenge test; GDM, gestational diabetes; NA, not applicable.

^a^ Index date is date of medication therapy initiation.

^b^ These are diagnoses occurring before the index date.

^c^ Based on the Carpentar and Coustan criteria^13,14^ or 1891 individuals with a GCT of 180 mg/dL or greater.

^d^ Overall, 56 switched from glyburide only to metformin and glyburide; 8 switched from glyburuide only to metformin, glyburide, and insulin; 587 switched from glyburide only to glyburide and insulin; and 9 switched from insulin only to insulin, glyburide, and metformin.

^e^ Overall, 24 switched from insulin only to metformin and insulin.

SE estimates were obtained using the influence curve^33^ of the inverse probability estimator for the coefficients of the linear marginal structural model. We used the missingness indicator approach^34,35,36,37^ to handle partially missing baseline and time-dependent covariate values (eTable 2 in the Supplement).

All analyses were conducted using R version 3.4.4 (R Project for Statistical Computing). Hypothesis tests were 2-sided. *P* < .05 was a priori considered significant.

## Results

Between January 2007 and December 2017, we identified 16 203 KPNC members with a singleton live birth who initiated medication therapy with either glyburide or insulin. We excluded pregnancies identified as being in the KPNC diabetes registry^38,39^ before (n = 2900) or during pregnancy (n = 370), those with no diagnosis of GDM (n = 34), or cases in which the medication therapy was initiated prior to the GDM diagnosis (n = 669). For individuals with multiple eligible pregnancies during the study period (n = 909), we included the first pregnancy in the study period. The final analytic cohort included 11 321 GDM pregnancies (Figure).

Of the 11 321 individuals with GDM who initiated glyburide or insulin during pregnancy (mean [SD] age, 32.9 [4.9] years), 10 249 (91%) used glyburide as the initial medication therapy, and 1072 (9%) used insulin as the initial medication therapy. Individuals who initiated glyburide as their initial medication therapy, compared with those who initiated insulin, were more likely to be Asian or Pacific Islander (3664 [36%] vs 277 [26%]), have normal weight (2190 [21%] vs 156 [15%]) or overweight (2708 [26%] vs 235 [22%]), and higher mean screening glucose values (mean [SD]. 173.4 [26.4] mg/dL vs 196.3 [57.2] mg/dL) (Table 1). The median (IQR) time from the index date to the analytic end of follow-up due to delivery was 7.0 (5.0-11.0) weeks for individuals receiving glyburide and 8 (5.0-15.0) weeks for individuals receiving insulin. Overall, 8845 individuals (86%) with glyburide as their initial medication therapy continued exclusive glyburide therapy until delivery, while 707 (66%) with insulin as their initial medication therapy continued exclusive insulin therapy until delivery.

### Perinatal and Neonatal Outcomes

After adjusting for index year, mother’s age at index, prepregnancy body mass index (BMI; calculated as weight in kilograms divided by height in meters squared), parity, race and ethnicity, gestational age at index, gestational age at GDM diagnosis, GDM duration at index, rate of weight gain, GCT screening value, depression, alcohol during pregnancy, smoking during pregnancy, illegal drug use during pregnancy, median household income, diastolic blood pressure, systolic blood pressure, preexisting hypertension, gestational hypertension, preeclampsia, hypertension medications, hypoglycemia, and met glycemic control, the risk of neonatal respiratory distress was 2.03 (95% CI, 0.13-3.92) per 100 births lower and the risk of NICU admission was 3.32 (95% CI, 0.20-6.45) per 100 births lower after continuous exposure to glyburide compared with insulin (Table 2). There were no statistically significant differences by initial medication therapy in risk for neonatal hypoglycemia (0.85 [95% CI, −1.17 to 2.86] per 100 births), jaundice (0.02 [95% CI, −1.46 to 1.51] per 100 births), shoulder dystocia (−1.05 [95% CI, −2.71 to 0.62] per 100 births), or large-for–gestational age categories (−2.75 [95% CI, −6.31 to 0.80] per 100 births). Results without accounting for changes in medication therapy after initial medication therapy (ITT analyses) were generally attenuated compared with results accounting for these changes (Table 3).

**Table 2.** Risk Estimates and Differences for Neonatal and Perinatal Outcomes for Glyburide and Insulin Accounting for Changes in Medication Therapy After Initial Medication Therapy (Per-Protocol Analyses)^a^

Outcome	Risk (SE) per 100 births		Risk difference (95% CI) per 100 births
	Glyburide	Insulin	
**Neonatal outcomes**			
Neonatal hypoglycemia			
Crude	6.25 (0.3)	6.22 (0.9)	0.03 (−1.82 to 1.88)
Adjusted	6.34 (0.3)	5.49 (1.0)	0.85 (−1.17 to 2.86)
Jaundice			
Crude	2.00 (0.1)	1.27 (0.4)	0.73 (−0.15 to 1.60)
Adjusted	2.04 (0.2)	2.01 (0.7)	0.02 (−1.46 to 1.51)
Shoulder dystocia			
Crude	2.44 (0.2)	3.82 (0.7)	−1.38 (−2.83 to 0.07)
Adjusted	2.48 (0.2)	3.53 (0.8)	−1.05 (−2.71 to 0.62)
Respiratory distress			
Crude	2.86 (0.2)	5.09 (0.8)	−2.23 (−3.89 to −0.57)
Adjusted	2.89 (0.2)	4.91 (0.9)	−2.03 (−3.92 to −0.13)
NICU admission			
Crude	10.00 (0.3)	14.37 (1.3)	−4.37 (−7.06 to −1.68)
Adjusted	10.19 (0.3)	13.51 (1.6)	−3.32 (−6.45 to −0.20)
**Size for gestational age**			
Small-for–gestational age			
Crude	7.46 (0.3)	8.49 (1.0)	−1.02 (−3.15 to 1.10)
Adjusted	7.44 (0.3)	8.64 (1.3)	−1.20 (−3.87 to 1.46)
Appropriate-for–gestational age			
Crude	75.66 (0.5)	70.16 (1.7)	5.50 (2.01 to 8.99)
Adjusted	75.26 (0.5)	71.31 (2.0)	3.95 (−0.15 to 8.06)
Large-for–gestational age			
Crude	16.88 (0.4)	21.36 (1.5)	−4.48 (−7.60 to −1.36)
Adjusted	17.30 (0.4)	20.05 (1.8)	−2.75 (−6.31 to 0.80)
**Maternal outcomes**			
Cesarean delivery			
Crude	38.65 (0.5)	43.42 (1.9)	−4.77 (−8.56 to −0.98)
Adjusted	38.76 (0.5)	40.68 (2.2)	−1.92 (−6.39 to 2.55)

Abbreviations: BMI, body mass index; GDM, gestational diabetes; NICU, neonatal intensive care unit.

^a^ Adjusted for index year, maternal age at index, prepregnancy body mass index, parity, race and ethnicity, gestational age at index, gestational age at gestational diabetes diagnosis, gestational diabetes duration at index, rate of weight gain, glucose challenge test, depression, alcohol during pregnancy, smoking during pregnancy, illegal drug during pregnancy, median household income, diastolic blood pressure, systolic blood pressure, preexisting hypertension, gestational hypertension, preeclampsia, hypertension medications, hypoglycemia, and met glycemic control (eTable 1 in the Supplement).

**Table 3.** Risk Estimates and Differences for Neonatal and Perinatal Outcomes for Glyburide and Insulin Treatment Without Accounting for Changes in Medication Therapy After Initial Medication Therapy (Intention-to-Treat Analyses)

Outcome	Risk (SE) per 100 births		Risk difference (95% CI) per 100 births
	Glyburide	Insulin	
**Neonatal outcomes**			
Neonatal hypoglycemia			
Crude	6.06 (0.2)	5.97 (0.7)	0.09 (−1.40 to 1.58)
Adjusted^a^	6.14 (0.2)	5.59 (0.7)	0.55 (−0.98 to 2.07)
Jaundice			
Crude	2.05 (0.1)	1.21 (0.3)	0.84 (0.13 to 1.55)
Adjusted^a^	2.07 (0.1)	1.35 (0.4)	0.73 (−0.10 to 1.56)
Shoulder dystocia			
Crude	2.57 (0.2)	3.54 (0.6)	−0.98 (−2.13 to 0.17)
Adjusted^a^	2.59 (0.2)	3.32 (0.6)	−0.73 (−1.92 to 0.46)
Respiratory distress			
Crude	2.81 (0.2)	4.29 (0.6)	−1.48 (−2.74 to −0.23)
Adjusted^a^	2.86 (0.2)	4.04 (0.6)	−1.18 (−2.46 to 0.10)
NICU admission			
Crude	10.03 (0.3)	13.51 (1.1)	−3.48 (−5.63 to −1.32)
Adjusted^a^	10.22 (0.3)	11.76 (1.0)	−1.54 (−3.66 to 0.57)
**Size for gestational age**			
Small-for–gestational age			
Crude	7.34 (0.3)	8.49 (0.9)	−1.15 (−2.89 to 0.59)
Adjusted^a^	7.26 (0.3)	9.14 (1.0)	−1.87 (−3.87 to 0.13)
Appropriate-for–gestational age			
Crude	74.93 (0.4)	70.15 (1.4)	4.78 (1.92 to 7.65)
Adjusted^a^	74.64 (0.4)	72.14 (1.5)	2.50 (−0.49 to 5.49)
Large-for–gestational age			
Crude	17.73 (0.4)	21.36 (1.3)	−3.63 (−6.20 to −1.07)
Adjusted^a^	18.10 (0.4)	18.73 (1.2)	−0.63 (−3.17 to 1.91)
**Maternal outcomes**			
Cesarean delivery			
Crude	39.66 (0.5)	44.68 (1.5)	−5.02 (−8.14 to −1.90)
Adjusted^a^	39.91 (0.5)	43.66 (1.6)	−3.75 (−7.09 to −0.41)

Abbreviation: NICU, neonatal intensive care unit.

^a^ Adjusted for index year, maternal age at index, prepregnancy body mass index, parity, race and ethnicity, gestational age at index, gestational age at gestational diabetes diagnosis, gestational diabetes duration at index, baseline rate of weight gain, glucose challenge test, depression, alcohol during pregnancy, smoking during pregnancy, illegal drug during pregnancy, median household income, baseline diastolic blood pressure, baseline systolic blood pressure, preexisting hypertension, baseline gestational hypertension, baseline preeclampsia, baseline hypertension medications, baseline hypoglycemia, and baseline met glycemic control (eTable 1 in the Supplement).

### Maternal Outcome

There was no difference in risk of cesarean delivery for continuous glyburide compared with insulin exposure (risk difference, −1.92 [95% CI, −6.39 to 2.55] per 100 births) (Table 2). The risk of cesarean delivery was significantly lower for exposure to glyburide compared with insulin in ITT analyses not accounting for changes in medication therapy after initial medication therapy (Table 3).

## Discussion

Using clinical data from an integrated health care delivery system with centralized GDM management and comprehensive information on glycemic control after GDM diagnosis, we did not find evidence of a difference in the perinatal outcomes examined for glyburide compared with insulin as first-line medication therapy. In fact, continuous use of glyburide was associated with decreased risk of neonatal respiratory distress and NICU admission compared with continuous use of insulin in analyses accounting for changes in medication therapy.

A recent meta-analysis of randomized clinical trials and observational studies comparing perinatal outcomes between glyburide and insulin as first-line medication therapy for GDM reported that glyburide therapy increased the risk of neonatal hypoglycemia, respiratory distress syndrome, birth injuries, reduced 5-minute Apgar score, and prolonged NICU admission duration overall, but when limited to randomized clinical trials, only associations with increased risk of neonatal hypoglycemia and prolonged NICU admission remained.^8^ However, prior observational studies^40^ did not adequately control for important confounders, such as GDM severity and BMI.

Previous randomized clinical trials comparing glyburide and insulin for GDM treatment have been criticized for incorrect dosing of glyburide and for allowing greater flexibility of the insulin regimen than for the glyburide regimen.^41,42^ In addition, almost all prior randomized trials had the primary outcome of maternal glycemia and were not powered to look at perinatal outcomes.^8^ Although initially thought not to cross the placenta, it has been confirmed that glyburide does cross the placenta, and fetal glyburide concentrations decrease rapidly over the 24-hour period after glyburide intake.^43^ There may have been differences in dose and timing of glyburide intake before delivery that may explain differences in observed neonatal hypoglycemia risk in our study compared with meta-analysis results.

Although most individuals in our study (91%) used glyburide as first-line therapy for GDM, reflecting the policy at the time within our health care system, individuals with indications of greater GDM severity (ie, prepregnancy obesity and higher 50-g OGTT screening values) were more likely to use insulin as first-line therapy. This underscores the importance of accounting for differences in GDM severity when comparing perinatal outcomes between glyburide and insulin treatment for GDM to mitigate bias concerns over unmeasured confounding. Very few individuals switched from glyburide to insulin in our study, highlighting patient preference for glyburide for its ease of use. Our use of inverse probability weighting accounted for baseline differences in GDM severity and other time-independent confounders as well as time-dependent confounders when accounting for changes in medication therapy after GDM medication therapy initiation.

Currently, insulin is recommended as first-line medication for treatment of GDM by the American Diabetes Association (ADA)^44^ and the American College of Obstetrics and Gynecologists (ACOG),^14^ and ACOG recommends that oral agents be reserved for those patients who refuse insulin and that glyburide should be avoided. However, given the limitations of past randomized trials on the topic and the advantages of oral agents over insulin for treatment of GDM, including greater ease of use and acceptance among patients, more well-designed studies that include information on longer-term safety outcomes for pregnant individuals and children are urgently needed.

### Strengths and Limitations

The strengths of our study include our use of modern causal inference methods combined with machine learning to emulate trial inferences using high-quality clinical data from all pregnant women with GDM treated with glyburide or insulin over a 10-year period in an integrated health care delivery system with centralized GDM management. Our analyses accounted for GDM severity, which often drives GDM treatment decisions, and changes in medication type over the course of treatment, which are common in individuals who have difficulty achieving adequate glycemic control. Although we accounted for many important confounders in our analyses using rigorous statistical methods, as with all observational studies, there remains the possibility of unmeasured confounding in our study. A randomized clinical trial is not feasible in our integrated health care delivery system given the high patient preference for oral agents and the additional patient burden associated with insulin use. At the time of this study was initiated we did not have a substantial number of patients with GDM initiated on metformin. It is important for future studies to compare glyburide with metformin given that both oral agents have the advantage of ease of use for patients.

This study also has limitations. A limitation of our implementation of the trial emulation methodology is the exclusion of women from our cohort based on events collected after the index date: mother’s death, disenrollment from the health plan, diabetes diagnosis, or end of pregnancy due to stillbirth. Such exclusions can lead to collider bias.^45^ However, with the exception of disenrollment from the health plan, these events are extremely rare, which mitigates the concern, and it is unlikely that disenrollment from the health plan and the outcomes studied share a common ancestor in a directed acyclic graph. Another limitation of our approach is that we only aimed to evaluate the effect of choice of drugs (ignoring exposure dose). The resulting estimates might thus be explained by differences in drug exposure dose.

## Conclusions

Our findings do not provide evidence of a difference in perinatal outcomes between glyburide users compared with insulin users. Given the current guidelines of the ADA and the ACOG, glyburide is not recommended as a first-line medication for the treatment of GDM. However, future work that examines long-term safety outcomes for women and children exposed to the different treatment regimens (glyburide, insulin, and metformin) for GDM is urgently needed.

## Notes

Supplement. **eTable 1.** List of Covariates Considered in the Analyses and Whether They Are Assumed to Be Associated With Outcomes or Exposure Decisions **eTable 2.** Cutoff Values Used to Discretize Continuous Covariates **eTable 3.** Super Learner Estimator for the Probability of Glyburide Initiation in the First 7 Days After Study Entry Given Baseline Covariates for Per-Protocol Analysis **eTable 4.** Super Learner Estimator for the Probability of Insulin Initiation in the First 7 Days After Study Entry Given Baseline Covariates Among Patients Who Did Not Initiate Glyburide at Study Entry for Per-Protocol Analysis **eTable 5.** Super Learner Estimator for the Probability of Continuing Glyburide After the First Follow-up Interval Given Past Covariates Among Patients Who Were Continuously Exposed to Glyburide Previously for Per-Protocol Analysis **eTable 6.** Super Learner Estimator for the Probability of Continuing Insulin After the First Follow-up Interval Given Past Covariates Among Patients Who Were Continuously Exposed to Insulin Previously for Per-Protocol Analysis **eTable 7.** Super Learner Estimator for the Probability of Glyburide Initiation on Index Date Given Baseline Covariates **eTable 8.** List of Covariates With Counts and Proportions of Missing Values at Baseline
