# Errors in Abstract, Table, and Article Information

**DOI:** 10.1001/jamanetworkopen.2022.12571

**Published:** 2022-04-21

In the Original Investigation titled “Association of Glyburide and Subcutaneous Insulin With Perinatal Complications Among Women With Gestational Diabetes,”^1^ published March 31, 2022, there were errors in the Abstract, Table 3, and Article Information. In the Abstract, the dates for the data analysis period should have read March 2017 to July 2019. In Table 3, the footnote should have indicated that the models were adjusted for baseline depression. In the Article Information, Dr Regenstein’s affiliation should have been The Permanente Medical Group, San Francisco, California. This article has been corrected.^1^
