# Supplementary material for: Association of Glyburide and Subcutaneous Insulin With Perinatal Complications Among Women With Gestational Diabetes
Source: JAMA Netw Open. 2022 Mar 31;5(3):e225026. doi: 10.1001/jamanetworkopen.2022.5026 (PMC8972026; doi:10.1001/jamanetworkopen.2022.5026)
Supplement: Supplement. — eTable 1. List of Covariates Considered in the Analyses and Whether They Are Assumed to Be Associated With Outcomes or Exposure Decisions eTable 2. Cutoff Values Used to Discretize Continuous Covariates eTable 3. Super Learner Estimator for the Probability of Glyburide Initiation in the First 7 Days After Study Entry Given Baseline Covariates for Per-Protocol Analysis eTable 4. Super Learner Estimator for the Probability of Insulin Initiation in the First 7 Days After Study Entry Given Baseline Covariates Among Patients Who Did Not Initiate Glyburide at Study Entry for Per-Protocol Analysis eTable 5. Super Learner Estimator for the Probability of Continuing Glyburide After the First Follow-up Interval Given Past Covariates Among Patients Who Were Continuously Exposed to Glyburide Previously for Per-Protocol Analysis eTable 6. Super Learner Estimator for the Probability of Continuing Insulin After the First Follow-up Interval Given Past Covariates Among Patients Who Were Continuously Exposed to Insulin Previously for Per-Protocol Analysis eTable 7. Super Learner Estimator for the Probability of Glyburide Initiation on Index Date Given Baseline Covariates eTable 8. List of Covariates With Counts and Proportions of Missing Values at Baseline [file jamanetwopen-e225026-s001.pdf]

Supplemental Online Content

Hedderson MM, Badon SE, Pimentel N, et al. Association of glyburide and subcutaneous insulin with perinatal complications among women with gestational diabetes. *JAMA Netw Open.* 2022;5(3):e225026. doi:10.1001/jamanetworkopen.2022.5026

eTable 1. List of Covariates Considered in the Analyses and Whether They Are Assumed to Be Associated With Outcomes or Exposure Decisions

eTable 2. Cutoff Values Used to Discretize Continuous Covariates

eTable 3. Super Learner Estimator for the Probability of Glyburide Initiation in the First 7 Days After Study Entry Given Baseline Covariates for Per-Protocol Analysis

eTable 4. Super Learner Estimator for the Probability of Insulin Initiation in the First 7 Days After Study Entry Given Baseline Covariates Among Patients Who Did Not Initiate Glyburide at Study Entry for Per-Protocol Analysis

eTable 5. Super Learner Estimator for the Probability of Continuing Glyburide After the First Follow-up Interval Given Past Covariates Among Patients Who Were Continuously Exposed to Glyburide Previously for Per-Protocol Analysis

eTable 6. Super Learner Estimator for the Probability of Continuing Insulin After the First Follow-up Interval Given Past Covariates Among Patients Who Were Continuously Exposed to Insulin Previously for Per-Protocol Analysis

eTable 7. Super Learner Estimator for the Probability of Glyburide Initiation on Index Date Given Baseline Covariates

eTable 8. List of Covariates With Counts and Proportions of Missing Values at Baseline

This supplemental material has been provided by the authors to give readers additional information about their work.

© 2022 Hedderson MM et al. *JAMA Network Open.*

eTable 1. List of Covariates Considered in the Analyses and Whether They Are Assumed to Be Associated With Outcomes or Exposure Decisions

Covariate	Outcome	Exposure	Time-dependent	Description
Alcohol.in.Preg	1	1	N	Indicator variable for alcohol during pregnancy
Depression	1	1	Y	Indicator variable for depression
Depression.0	1	1	N	Indicator variable for depression (baseline)
Diastolic	1	1	Y	Diastolic blood pressure
Diastolic.0	1	1	N	Diastolic blood pressure (baseline)
GA.GDM.Diagnosis	1	1	N	Gestational age at diagnosis of GDM (in weeks)
GA.Index	1	1	N	Gestational age at index date (in weeks)
GDM.Diagnosis	1	1	N	GDM diagnosis from OGTT Carpenter & Coustan criteria or doctor
GDM.Duration.Index	1	1	N	Gestational diabetes duration at index
Gestational.HTN	1	1	Y	Indicator variable for gestational hypertension
Gestational.HTN.0	1	1	N	Indicator variable for gestational hypertension (baseline)
HTN.Rx	1	1	Y	Indicator variable for fills of hypertension medications
HTN.Rx.0	1	1	N	Indicator variable for fills of hypertension medications (baseline)
Hypoglacemia	1	1	Y	Indicator variable for hypoglycemia
Hypoglacemia.0	1	1	N	Indicator variable for hypoglycemia (baseline)
Hypoglacemia.behav.1	1	1	Y	Indicator variable for hypoglycemia
IllegalDrug.in.Preg	1	1	N	Indicator variable for illegal drugs during pregnancy
Index.Year	1	1	N	Year of study entry
Median.House.Income	1	1	N	Median household income
Met.Glycemic.Control	1	1	Y	Indicator variable for optimal glycemic control achieved
Met.Glycemic.Control.0	1	1	N	Indicator variable for optimal glycemic control achieved (baseline)
Mom.Age.Index	1	1	N	Maternal age at index date
Parity	1	1	N	Parity (not including the index pregnancy)
Preeclampsia	1	1	Y	Indicator variable for preeclampsia
Preeclampsia.0	1	1	N	Indicator variable for preeclampsia (baseline)
PreExisting.HTN	1	1	N	Indicator variable for pre-existing hypertension
Prepregnancy.BMI	1	1	N	Pre-pregnancy BMI
Race.Ethnicity	1	1	N	Maternal race/ethnicity
Rate.of.Wt.Gain	1	1	N	Rate of weekly weight gain at index
Rate.of.Wt.Gain.time.updated	1	1	Y	Rate of weekly weight gain after index
Screening.Value	1	1	N	50-g glucose Screening test result
Severity	1	1	N	Marker for GDM Screening Value≥140

© 2022 Hedderson MM et al. *JAMA Network Open.*

Smoking.in.Preg	1	1	N	Indicator variable for smoking during pregnancy
Systolic	1	1	Y	Systolic blood pressure
Systolic.0	1	1	N	Systolic blood pressure (baseline)

© 2022 Hedderson MM et al. *JAMA Network Open.*

eTable 2. Cutoff Values Used to Discretize Continuous Covariates

Variable	Cutoffs
Diastolic	80
Diastolic.0	80
GA.GDM.Diagnosis	24;29
GA.Index	26;33
GDM.Duration.Index	2;5;9
intnum	1;12;13;17;21;25
Median.House.Income	30000;50000;70000;90000
Mom.Age.Index	30;35;40
Parity	0;1;2
Prepregnancy.BMI	18.5; 25; 30; 35
Rate.of.Wt.Gain	0.05;.22;.31
Rate.of.Wt.Gain.time.updated	0.05;.22;.31
Screening.Value	140;155;200
Systolic	120
Systolic.0	120

© 2022 Hedderson MM et al. *JAMA Network Open.*

eTable 3. Super Learner Estimator for the Probability of Glyburide Initiation in the First 7 Days After Study Entry Given Baseline Covariates

Estimators derived based on 11321 observations from 11321 unique patients.Seven learners were considered: A logistic model (glm), splines (poly), a random forest (ranger), an xgboost (xgb), a generalized additive model (gam), a lasso model (glmnet), and a bayesian additive regression tree (bart), a logistic model with all two-way interaction terms (glm.int), a neural network regression with a single hidden layer (nnet). Screeners (i.e. feature selection denoted by 'Rank') were considered for all learner sand were defined by the top 30, 20, 10, and 5 variables most associated (i.e.,with smallest p-values in a univariate regression) with the exposure (A). The weighted average (SL weights) of these 45 learners that define the super learner is based on 10-fold cross-validation (CV).

	glm	glm Rank 5	glm Rank 10	glm Rank 20	glm Rank 30
CV risk	0.07814	0.08061	0.07924	0.07876	0.07869
SL weights	0	0	0	0	0

poly	poly Rank 5	poly Rank 10	poly Rank 20	poly Rank 30
0.07863	0.07991	0.07908	0.07887	0.07878
0.01695	0	0	0	0

rngr	rngr Rank 5	rngr Rank 10	rngr Rank 20	rngr Rank 30
0.07906	0.08043	0.07945	0.07993	0.07973
0.1292	0	0	0	0.10792

xgb	xgb Rank 5	xgb Rank 10	xgb Rank 20	xgb Rank 30
0.08449	0.08144	0.08094	0.0834	0.08355
0	0	0.0624	0	0.01412

gam	gam Rank 5	gam Rank 10	gam Rank 20	gam Rank 30
0.07802	0.08052	0.07901	0.07838	0.07834
0.47769	0	0	0	0

© 2022 Hedderson MM et al. *JAMA Network Open.*

glmnet	glmnet Rank 5	glmnet Rank 10	glmnet Rank 20	glmnet Rank 30
0.07811	0.08063	0.07926	0.07876	0.07857
0	0	0	0	0

bart	bart Rank 5	bart Rank 10	bart Rank 20	bart Rank 30
0.76667	0.76088	0.76412	0.76712	0.76665
0	0	0	0	0

glm.int	glm.int Rank 5	glm.int Rank 10	glm.int Rank 20	glm.int Rank 30
0.25899	0.08049	0.0791	0.07989	0.09816
8.49e-03	0	0.03601	0.14722	0

nnet	nnet Rank 5	nnet Rank 10	nnet Rank 20	nnet Rank 30
0.09743	0.09743	0.09743	0.09743	0.09743
0	0	0	0	0

eTable 4. Super Learner Estimator for the Probability of Insulin Initiation in the First 7 Days After Study Entry Given Baseline Covariates Among Patients Who Did Not Initiate Glyburide at Study Entry Estimators derived based on 1103 observations from 1103 unique patients. Seven learners and 4 screeners were considered (see caption of Appendix Table 3 for details).

	glm	glm Rank 5	glm Rank 10	glm Rank 20	glm Rank 30
CV risk	0.0412	0.02968	0.03005	0.03127	0.03191
SL weights	0	0	0	0	0

poly	poly Rank 5	poly Rank 10	poly Rank 20	poly Rank 30
0.02934	0.02934	0.02935	0.02935	0.02934
0	0	0	0	0

rngr	rngr Rank 5	rngr Rank 10	rngr Rank 20	rngr Rank 30
0.02986	0.03016	0.0303	0.03105	0.03049
0	0	0.02229	0	0

xgb	xgb Rank 5	xgb Rank 10	xgb Rank 20	xgb Rank 30
0.0304	0.0299	0.02984	0.03013	0.03025
0	0	0	0.04303	0

gam	gam Rank 5	gam Rank 10	gam Rank 20	gam Rank 30
0.04223	0.02966	0.02997	0.03129	0.03236
0	0	0.09886	0.06211	0

© 2022 Hedderson MM et al. *JAMA Network Open.*

glmnet	glmnet Rank 5	glmnet Rank 10	glmnet Rank 20	glmnet Rank 30
0.0292	0.02943	0.02967	0.0301	0.03004
0	0.13188	0	0	0

bart	bart Rank 5	bart Rank 10	bart Rank 20	bart Rank 30
0.91148	0.91253	0.91097	0.91187	0.91343
0	0.15695	0	0	0

glm.int	glm.int Rank 5	glm.int Rank 10	glm.int Rank 20	glm.int Rank 30
0.49608	0.03161	0.03359	0.11555	0.22591
0	0	0	0	0

nnet	nnet Rank 5	nnet Rank 10	nnet Rank 20	nnet Rank 30
0.02992	0.02992	0.02992	0.02992	0.02992
0.48488	0	0	0	0

© 2022 Hedderson MM et al. *JAMA Network Open.*

eTable 5. Super Learner Estimator for the Probability of Continuing Glyburide After the First Follow-up Interval Given Past Covariates Among Patients Who Were Continuously Exposed to Glyburide Previously

Estimators derived based on 83466 observations from 10042 unique patients. Seven learners and 4 screeners were considered (see caption of Appendix Table 3 for details).

	glm	glm Rank 5	glm Rank 10	glm Rank 20	glm Rank 30
CV risk	0.01559	0.01566	0.01564	0.01563	0.01563
SL weights	0	0	0	0	0

poly	poly Rank 5	poly Rank 10	poly Rank 20	poly Rank 30
0.01541	0.01541	0.01545	0.01546	0.01546
0.0564	0	0	0	0

rngr	rngr Rank 5	rngr Rank 10	rngr Rank 20	rngr Rank 30
0.0151	0.01541	0.01543	0.01527	0.01535
0.20883	0	0	0.07018	0.09879

xgb	xgb Rank 5	xgb Rank 10	xgb Rank 20	xgb Rank 30
0.01551	0.01537	0.01582	0.0155	0.01558
0.25095	0.05134	0	0.06975	0.03662

gam	gam Rank 5	gam Rank 10	gam Rank 20	gam Rank 30
0.01558	0.01566	0.01564	0.01563	0.01563
0	0	0	0	0

© 2022 Hedderson MM et al. *JAMA Network Open.*

glmnet	glmnet Rank 5	glmnet Rank 10	glmnet Rank 20	glmnet Rank 30
0.01558	0.01567	0.01565	0.01563	0.01563
0	0	0	0	0

bart	bart Rank 5	bart Rank 10	bart Rank 20	bart Rank 30
0.95494	0.95387	0.95385	0.95552	0.95546
0	0	0.13971	0	0

glm.int	glm.int Rank 5	glm.int Rank 10	glm.int Rank 20	glm.int Rank 30
0.32922	0.01539	0.01534	0.01554	0.11825
4.5e-04	0	0	0.01411	1.66e-03

nnet	nnet Rank 5	nnet Rank 10	nnet Rank 20	nnet Rank 30
0.01645	0.01645	0.01645	0.01645	0.01645
1.22e-03	0	0	0	0

© 2022 Hedderson MM et al. *JAMA Network Open.*

eTable 6. Super Learner Estimator for the Probability of Continuing Insulin After the First Follow-up Interval Given Past Covariates Among Patients Who Were Continuously Exposed to Insulin Previously

Estimators derived based on 8311 observations from 1041 unique patients. Seven learners and 4 screeners were considered (see caption of Appendix Table 3 for details).

	glm	glm Rank 5	glm Rank 10	glm Rank 20	glm Rank 30
CV risk	0.04203	0.04166	0.04156	0.04158	0.04171
SL weights	0	0	0	0	0

poly	poly Rank 5	poly Rank 10	poly Rank 20	poly Rank 30
0.03528	0.03536	0.03526	0.03533	0.03536
0.34827	0	0.22101	0	0

rngr	rngr Rank 5	rngr Rank 10	rngr Rank 20	rngr Rank 30
0.03987	0.03584	0.03644	0.03749	0.03859
0	0.03734	0	0.05356	0

xgb	xgb Rank 5	xgb Rank 10	xgb Rank 20	xgb Rank 30
0.04337	0.03594	0.03745	0.03917	0.04146
0.07854	0.1261	0	0.12776	0

gam	gam Rank 5	gam Rank 10	gam Rank 20	gam Rank 30
0.04166	0.04138	0.04127	0.0413	0.04139
0	0	0	0	0

© 2022 Hedderson MM et al. *JAMA Network Open.*

glmnet	glmnet Rank 5	glmnet Rank 10	glmnet Rank 20	glmnet Rank 30
0.04138	0.04165	0.04152	0.04148	0.04148
0	0	0	0	0

bart	bart Rank 5	bart Rank 10	bart Rank 20	bart Rank 30
0.88687	0.89308	0.89206	0.89243	0.89189
0	0	0	0	0

glm.int	glm.int Rank 5	glm.int Rank 10	glm.int Rank 20	glm.int Rank 30
0.5015	0.04193	0.04304	0.05007	0.18032
0	0	0	7.42e-03	0

nnet	nnet Rank 5	nnet Rank 10	nnet Rank 20	nnet Rank 30
0.04368	0.04368	0.04368	0.04368	0.04368
0	0	0	0	0

© 2022 Hedderson MM et al. *JAMA Network Open.*

eTable 7. Super Learner Estimator for the Probability of Glyburide Initiation on Index Date Given Baseline Covariates

Estimators derived based on 11321 observations from 11321 unique patients. Seven learners and 4 screeners were considered (see caption of Appendix Table 3 for details).

	glm	glm Rank 5	glm Rank 10	glm Rank 20	glm Rank 30
CV risk	0.07604	0.07853	0.07713	0.07685	0.07658
SL weights	0	0	0	0	0

poly	poly Rank 5	poly Rank 10	poly Rank 20	poly Rank 30
0.07639	0.07782	0.07692	0.07685	0.07661
0.07399	0	0	0	0

rngr	rngr Rank 5	rngr Rank 10	rngr Rank 20	rngr Rank 30
0.07702	0.07838	0.07734	0.07823	0.07781
0.18527	0	0	0	1.17e-03

xgb	xgb Rank 5	xgb Rank 10	xgb Rank 20	xgb Rank 30
0.08216	0.0793	0.07866	0.08131	0.08211
3.86e-03	0	0.09829	2.5e-03	0

gam	gam Rank 5	gam Rank 10	gam Rank 20	gam Rank 30
0.07595	0.07845	0.07693	0.07649	0.07625
0.45177	0	0	0	0

© 2022 Hedderson MM et al. *JAMA Network Open.*

glmnet	glmnet Rank 5	glmnet Rank 10	glmnet Rank 20	glmnet Rank 30
0.07595	0.07855	0.07716	0.07688	0.07653
0	0	0	0	0

bart	bart Rank 5	bart Rank 10	bart Rank 20	bart Rank 30
0.77292	0.76711	0.77049	0.77277	0.77258
0	0	0	0	0

glm.int	glm.int Rank 5	glm.int Rank 10	glm.int Rank 20	glm.int Rank 30
0.26349	0.07842	0.07703	0.07797	0.11557
4.34e-03	0	0	0.15631	0.02249

nnet	nnet Rank 5	nnet Rank 10	nnet Rank 20	nnet Rank 30
0.09469	0.09469	0.09469	0.09469	0.09469
0	0	0	0	0

© 2022 Hedderson MM et al. *JAMA Network Open.*

**eTable 8.** List of Covariates With Counts and Proportions of Missing Values at Baseline		
Covariate	N_Missing values	%_Missing values
Depression.0	0	0.0%
Diastolic.0	192	1.7%
Gestational.HTN.0	0	0.0%
HTN.Rx.0	0	0.0%
Hypoglacemia.0	0	0.0%
Median.House.Income	93	0.8%
Met.Glycemic.Control.0	1031	9.1%
Mom.Age.Index	0	0.0%
Parity	160	1.4%
Preeclampsia.0	0	0.0%
PreExisting.HTN	0	0.0%
Prepregnancy.BMI	654	5.8%
Race.Ethnicity	20	0.2%
Rate.of.Wt.Gain	940	8.3%
Severity	971	8.6%
Systolic.0	192	1.7%

© 2022 Hedderson MM et al. *JAMA Network Open.*
